# The Effect of the Potential PhoQ Histidine Kinase Inhibitors on *Shigella flexneri* Virulence

**DOI:** 10.1371/journal.pone.0023100

**Published:** 2011-08-10

**Authors:** Xia Cai, Jian Zhang, Mingliang Chen, Yang Wu, Xueqing Wang, Jiayu Chen, Junqin Zhang, Xu Shen, Di Qu, Hualiang Jiang

**Affiliations:** 1 Key Laboratory of Medical Molecular Virology of Ministries of Education and Health, Institute of Medical Microbiology and Institutes of Biomedical Sciences, Fudan University, Shanghai, China; 2 Drug Discovery and Design Center, State Key Laboratory of Drug Research, Shanghai Institute of Materia Medica, Shanghai Institutes for Biological Sciences, Graduate School of the Chinese Academy of Sciences, Chinese Academy of Sciences, Shanghai, China; 3 Shanghai Municipal Center for Disease Control and Prevention, Shanghai, China; East Carolina University School of Medicine, United States of America

## Abstract

PhoQ/PhoP is an important two-component system that regulates *Shigella* virulence. We explored whether the PhoQ/PhoP system is a promising target for new antibiotics against *S. flexneri* infection. By using a high-throughput screen and enzymatic activity coupled assay, four compounds were found as potential PhoQ inhibitors. These compounds not only inhibited the activity of SF-PhoQc autophosphorylation but also displayed high binding affinities to the SF-PhoQc protein in the Surface Plasmon Resonance response. A *S. flexneri* cell invasion assay showed that three of these potential PhoQ inhibitors inhibit the invasion of HeLa cells by *S. flexneri* 9380. In a Mouse Sereny test, mice inoculated with *S. flexneri* 9380 pre-treated with the potential PhoQ inhibitors 1, 2, 3 or 4 displayed no inflammation, whereas mice inoculated with *S. flexneri* 9380 alone displayed severe keratoconjunctival inflammation. All four potential PhoQ inhibitors showed no significant cytotoxicity or hemolytic activity. These data suggest that the four potential PhoQ inhibitors inhibited the virulence of *S. flexneri* and that PhoQ/PhoP is a promising target for the development of drugs against *S. flexneri* infection.

## Introduction

*Shigella* is a gram-negative facultative intracellular pathogen with enhanced cell invasion, intracellular growth and intercellular spreading capabilities. The bacteria are transmitted fecal-orally and will invade the mucosa of the colon. Infection by only 10 to 100 organisms will cause shigellosis. Because of the overuse of antibiotics, *Shigella* drug resistance in clinical settings is increasing [1], [2], [3]. Therefore, new therapeutic targets and drugs are needed to reduce the incidence of shigellosis worldwide. Understanding the regulation of *Shigella* virulence may lead to the development of new drugs that can inhibit or reduce the virulence of *Shigella* as well as provide new strategies for treating shigellosis.

PhoQ/PhoP is a two-component system (TCS) that governs virulence, monitors extracellular Mg^2+^, and regulates several cellular activities in many gram-negative species [4]. The PhoQ/PhoP TCS consists of the transmembrane sensor PhoQ and the cytoplasmic regulator PhoP. PhoQ is a transmembrane histidine kinase with a functional kinase domain that binds ATP. It responds to environmental signals by phosphorylating itself as well as PhoP. PhoP has a functional domain, which when phosphorylated influences virulence by activating a phosphorylation cascade that regulates a series of downstream effecter genes in several bacterial species, including *Shigella flexneri*, *Salmonella enterica*, and *Escherichia coli* [5], [6]. In *Shigella*, a functional *phoP* gene is important for virulence [7]. It has been proven that PhoP regulates *Shigella*'s susceptibility to polymorphonuclear leucocytes (PMNs) and antimicrobial molecules. A *phoP Shigella* mutant is highly sensitive to killing by neutrophils [7]. Furthermore, infection of a mouse eye with a wild-type *Shigella* strain will cause keratoconjunctivitis, whereas infection by a *phoP Shigella* mutant was resolved more quickly relative to wild type infections [7]. The research of PhoQ/PhoP TCS in *Salmonella* showed that mutants in the PhoQ/PhoP system can greatly reduce bacterial virulence and intracellular survival in macrophages [8]. This prompted us to investigate whether PhoQ/PhoP in *Shigella* would be an appropriate target for the design of novel antibacterial agents.

In the present study, we chose the PhoQ protein of *S. flexneri* as the target for screening by a chemical library, and four potential PhoQ inhibitors were identified. Both the cell invasion assay and Mouse Sereny test showed that these potential PhoQ inhibitors abate the virulence of *S. flexneri.* These potential PhoQ inhibitors displayed low cytotoxicity on mammalian cells and had no hemolysis effect. Our data indicate that PhoQ may be a promising target for the development of new antibiotics to treat *S. flexneri* infection.

## Results

### Sequence analysis of PhoQ HK and structure-based discovery of potential PhoQ inhibitors *in silico*

**Figure 1:**
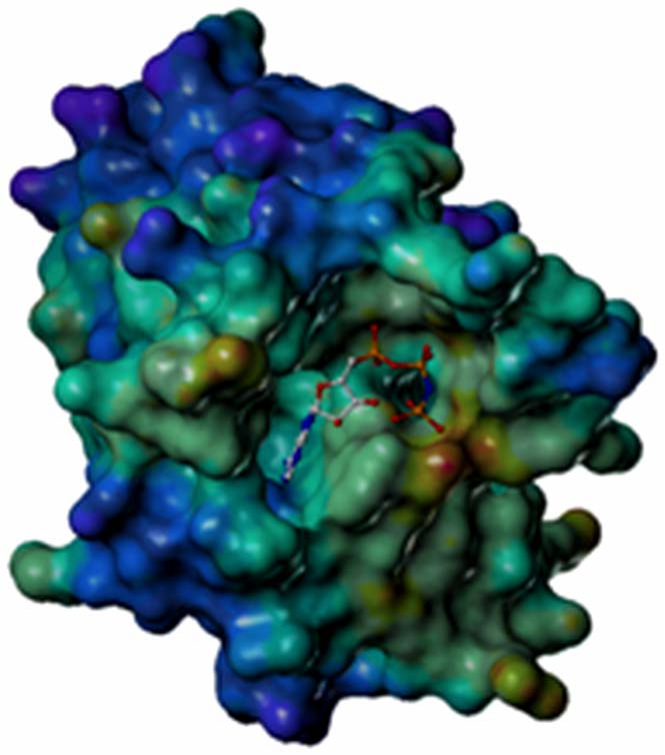
Shape and surface features of the ATP-binding pocket of the PhoQ HK domain. The 3D model of the PhoQ HK domain of *Sf*301 shown was constructed based on the crystal structure of the PhoQ HK of *E. coli*, which is highly homologous to *S. flexneri* (more than 99.8%). The ATP binding pocket is fairly large and deep. Two cavities joined by a gorge-like channel create the whole binding pocket.

Alignment of PhoQ amino acid sequences of the *Shigella* strains *S. flexneri* 301, *S. dysenteriae* 197, *S. boydii* 227 and *S. sonnei* 046 show that the proteins share 100% identity. An optimized 3D model of the PhoQ HK domain of *Sf*301 was constructed based on the crystal structure of PhoQ HK from *E .coli*, which is highly homologous to *S. flexneri* (more than 99.8%). The ATP binding site consists of two different cavities connected by a gorge-like channel (Figure 1). The 3D model was used to identify potential PhoQ inhibitors of *S. flexneri* PhoQ HK by high-throughput virtual screening (HTVS). Using the drug-selection filter from the SPECS database, 85,000 potential drug-like molecules, constituting an in-house database (named SPECS_1), were searched for potential binding molecule structures, using the program DOCK4.0 as a primary screening. Then, the most optimal 200 structures were rescored with the programs FlexX and CSCORE. Finally, according to molecular diversity, shape complementarity, and potential for forming hydrogen bonds in the binding pocket of the PhoQ, 100 molecules were manually selected as inhibitor candidates, and 64 compounds were purchased from the SPECS Company for further bioassays.

### Effect of the potential PhoQ inhibitors on enzymatic activities of SF-PhoQc

To measure the interaction of the 64 compounds with PhoQ, a prokaryotic expression plasmid containing the *Sf3*01 PhoQ intracellular domain was constructed, the recombinant His-tagged SF-PhoQc was purified and analyzed by SDS-PAGE (Figure S1A), and western blot analysis (Figure S1B) was performed with a mouse anti-His monoclonal antibody. The molecular mass of the recombinant SF-PhoQc was confirmed by MALDI-MS as 27.914 kDa (Figure S1C), which is in agreement with the theoretical molecular mass of 27.914 kDa.

**Figure 2:**
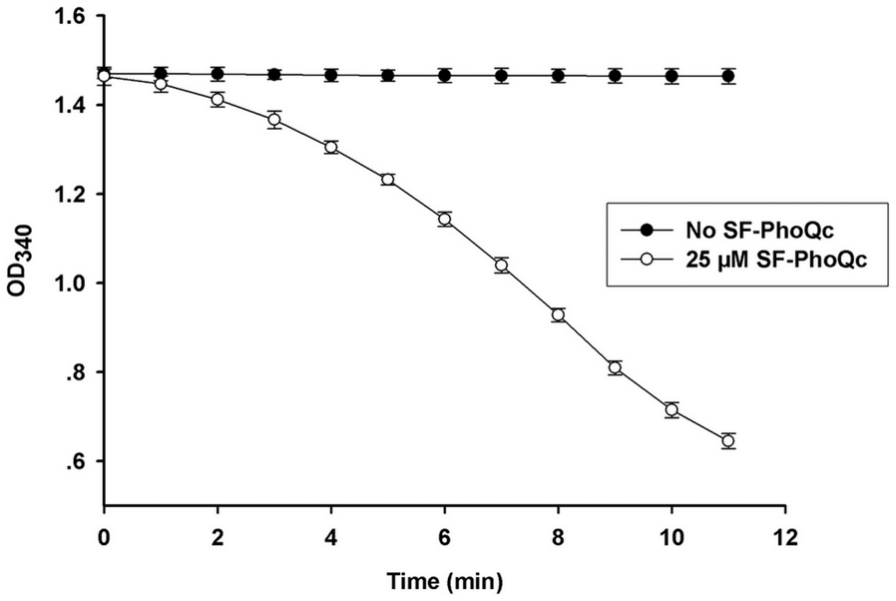
The activity of recombinant SF-PhoQc. The activity of SF-PhoQc from the Pyrophosphate Reagent was shown. All assays were conducted in a 96-well microplate spectrophotometer. The assay mixture (total volume was 200 µl) contained 25 mmol/L KCl, 2.5 mmol/L MgCl_2_, 2 mmol/L ATP, 1 mmol/L phosphoendpyruvate, 0.35 mmol/L NADH, 1.5 U/ml PK, 1.25 U/ml LDH and 50 mmol/L Tris-Cl (pH 8.0). Protein SF-PhoQc was added to a final concentration of 25 µmol/L to start the reaction. After SF-PhoQc was added, the A_340_ value was recorded by SoftMax vision 4.8 every minute for 30 minutes.

The enzymatic activity of SF-PhoQc was determined using the Pyrophosphate Reagent, in which ATP initiates the reaction and ADP generated by ATP is accompanied by the reduction of NADH to NAD^+^. The rate of NADH reduction to NAD^+^ is detected by a change in absorbance readings at 340 nm. The activity of SF-PhoQc is measured by detecting a decrease in NADH at A_340_. Recombinant SF-PhoQc (25 µmol/L) was added to initiate the reaction, and the A_340_ value was recorded by SoftMax vision 4.8 each minute for 30 minutes. The A_340_ value reduced from 1.47 to 0.65 in the presence of SF-PhoQc (Figure 2).

**Figure 3:**
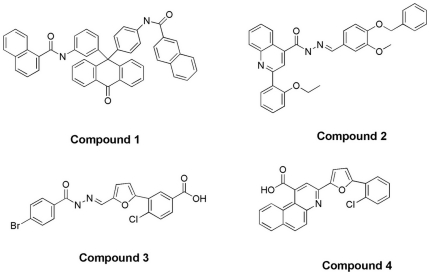
The chemical structures of four compounds as potential PhoQ inhibitors.

**Figure 4:**
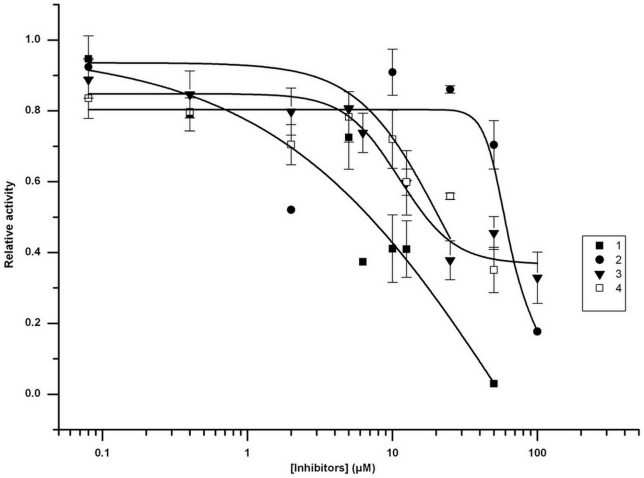
Dose response curve of recombinant SF-PhoQc enzyme inhibition by potential PhoQ inhibitors 1–4. The inhibition effects of potential PhoQ inhibitors on SF-PhoQc at different concentrations (0–100 µmol/L) detected by using the Pyrophosphate Reagent, and the dose-dependent curve of each compound were measured by using Origin 7.0 software (OriginLab, Northampton, USA). Relative activity in Y-axis was calculated by 100% –Rp, indicating the enzyme activity of SF-PhoQc after potential PhoQ inhibitor treatment.

The effect of the 64 compounds on SF-PhoQc enzymatic activity was measured by using Pyrophosphate Reagent (Sigma-Aldrich, MO, USA). At a concentration of 100 mmol/L, four compounds (1, 2, 3 and 4) (Figure 3) inhibited the enzymatic activity of SF-PhoQc. Compounds 1, 2, 3 and 4 decreased SF-PhoQc catalyzed consumption of ATP by 100%, 82.3%, 67.1% and 65.8%, respectively (Figure 4). Then, the IC_50_ of each compound was determined by adding 4 µg of purified SF-PhoQc to different concentrations of each compound. The IC_50_ values of compounds 1–4 were 12.03±3.91 µmol/L, 56.99±1.04 µmol/L, 27.77±10.70 µmol/L and 27.75±5.59 µmol/L, respectively (Table 1).

**Table 1:** Biological effects of the four potential PhoQ inhibitors.

Potential PhoQ inhibitors No.	IC_50_ (µmol/L) for SF-PhoQc(a)	IC_50_ (µmol/L) for SF-PhoQc(b)	K_D_ value (µmol/L)(c)	CC_50_ (µmol/L) on Vero cell(d)	Hemolysis (%)
1	12.03±3.91	69.37±0.56	4.50	>200	<0.1(e) (<0.1(f))
2	56.99±1.04	48.90±14.7	10.6	>200	<0.1 (<0.1)
3	27.77±10.70	7.99±5.61	7.56	>200	<0.1 (<0.1)
4	27.75±5.59	27.20±15.44	9.40	>200	<0.1 (<0.1)

(a) The Pyrophosphate Reagent detects autophosphorylation. IC_50_ represents the concentration of the potential PhoQ inhibitor needed to inhibit 50% of SF-PhoQc's autophosphorylation. Values are means ± standard deviations from 3 independent wells.

(b) The Luminescent Kinase Assay detects autophosphorylation. IC_50_ represents the concentration of the potential PhoQ inhibitor needed to inhibit 50% of SF-PhoQc's autophosphorylation. Values are means ± standard deviations from 3 independent wells.

(c) K_D_: Equilibrium Dissociation constant.

(d) CC_50_ represents the concentration of a potential PhoQ inhibitor that results in 50% cytotoxicity of Vero cells. The highest concentration tested is 200 µmol/L.

(e) Healthy human erythrocytes were used for the hemolysis assay, and the hemolytic activities of the four potential PhoQ inhibitors are shown at (e)25 µmol/L and

(f) 100 µmol/L.

Luminescent Kinase Assay was used to confirm the effects of compounds 1, 2, 3 and 4 on the enzymatic activity of SF-PhoQc. In the Luminescent Kinase Assay, the putative kinase activity of SF-PhoQc protein was measured by quantifying the amount of ATP that remained in solution after the reaction. A direct relationship between the luminescence measured and the amount of ATP was observed, indicating that the purified SF-PhoQc protein possessed ATPase activity. Next, the effect of each compound on the enzymatic activity of SF-PhoQc was measured. The IC_50_ values of compounds 1–4 were calculated as 69.37±0.56 µmol/L, 48.9±14.7 µmol/L, 7.99±5.61 µmol/L and 27.2±15.44 µmol/L, respectively (Table 1).

### Binding affinities of potential PhoQ inhibitors to SF-PhoQc

**Figure 5:**
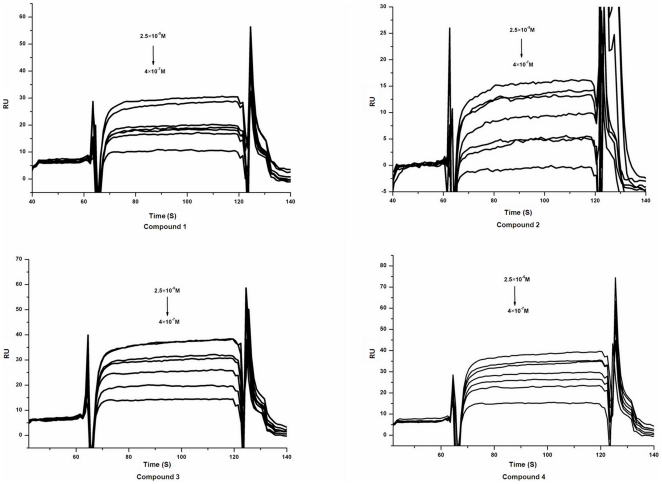
Binding affinities of the potential PhoQ inhibitors to the SF-PhoQc protein determined by using SPR. Real-time measurements of the interactions of potential PhoQ inhibitors 1–4 with the SF-PhoQc protein using the Biacore 3000 instrument were shown. The curves represent the interaction of various concentrations of compounds with the protein. The potential PhoQ inhibitors were injected for 120 seconds, and dissociation was monitored for more than 150 seconds.

The binding affinities of compounds 1–4 to the SF-PhoQc were determined by Surface Plasmon Resonance (SPR). All four compounds displayed significant binding affinities to the SF-PhoQc protein in the SPR response (Figure 5). The data were fitted to a steady-state affinity model for potential PhoQ inhibitors, and a K_D_ value was determined using the Biacore 3000 evaluation software. The dissociation constants of compounds 1–4 to SF-PhoQc were 4.50, 10.6, 7.56 and 9.40 µmol/L, respectively, and they were named as potential PhoQ inhibitors (Table 1).

### Evaluation of potential PhoQ inhibitors on the virulence of *S. flexneri* and *S. typhimurium in vitro*

**Figure 6:**
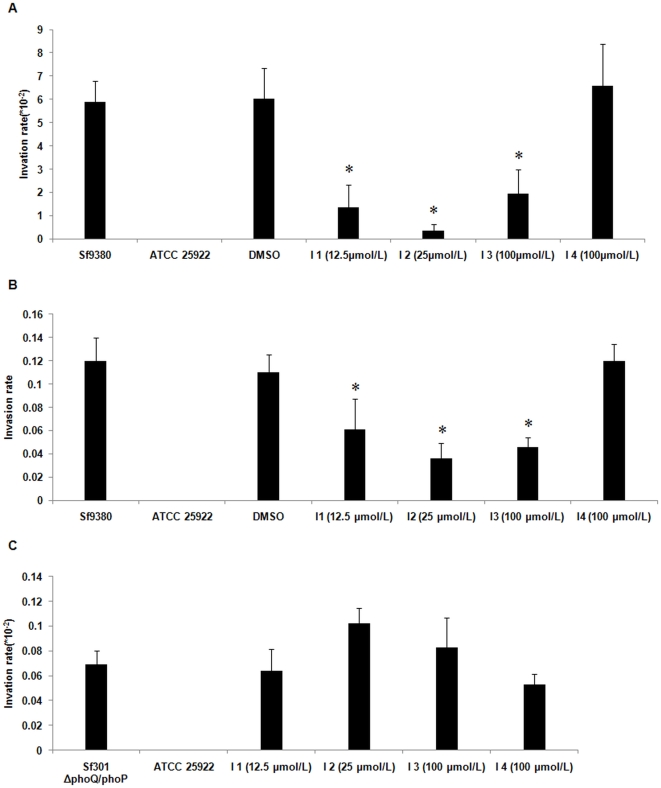
The effects of potential PhoQ inhibitors on virulence of *Shigella*. The gentamicin protection assay shows the inhibitory effect of the four potential PhoQ inhibitors on *S. flexneri* HeLa cells invasion. The bacteria pre-treated with potential PhoQ inhibitor were added to HeLa cells at a multiplicity of infection of 100. Then gentamicin was added to kill extracellular bacteria and cell lysates were plated onto LB plates, and the colonies were counted. The results of the assays were expressed as the number of bacteria recovered from gentamicin-treated cells divided by the number of inoculated bacteria added to the cell. *S. flexneri Sf*9380 (treated without DMSO), a clinical strain with strong virulence, served as the positive control for the cell invasion assay; *E. coli* ATCC 25922 (treated without DMSO), an avirulent strain, served as the negative control for the cell invasion assay; bacteria treated with 0.1% DMSO (the solvent of the potential PhoQ inhibitor) also served as a control for the cell invasion assay. Values are means ± standard deviations from 6 independent wells. *p<0.01 vs. *Sf*9380. (**A**) The effects of potential PhoQ inhibitors on cell invasion of *S. flexneri Sf*9380 (**B**) The effects of potential PhoQ inhibitors on intracellular growth *S. flexneri Sf*9380. (**C**) The effects of potential PhoQ inhibitors on cell invasion of *S. flexneri 2a* 301 *phoQ/phoP* knock-out mutant.

At concentration of 200 µmol/L (highest concentration in the present study) the four potential PhoQ inhibitors had no obvious effect on growth of *Sf*9380. The growth curve of *Sf*9380 treated with or without potential PhoQ inhibitors at concentration 200 µmol/L were tested. The growth curves of bacteria treated with or without potential PhoQ inhibitors treatment were similar, suggesting that four potential PhoQ inhibitors have no effects on the growth of *Sf*9380 (Figure S2). The gentamicin protection assay was used as a cellular model to evaluate the inhibitory effect of the four potential PhoQ inhibitors [9]. The bacteria were treated with each of the four potential PhoQ inhibitors in a serial dilution and grown for 8 hours. Next, the bacteria were inoculated and incubated with HeLa cells for 60 min prior to the addition of gentamicin to kill extracellular bacteria. For testing intracellular growth and spreading to adjacent cell, cells were further incubated for 290 min [9]. The results of the assays were expressed as the number of bacteria recovered from gentamicin-treated cells divided by the number of inoculated bacteria added to the cell. After lysates of HeLa cells were plated, the number of CFU that formed on LB agar were counted. Treated with 12.5 µmo/L of potential PhoQ inhibitor 1, invasion of HeLa cells by *Sf*9380 was reduced to 1.36×10^−2^, 4.3 times lower than that of untreated *Sf*9380 (5.87×10^−2^). A concentration of 25 µmo/L of potential PhoQ inhibitor 1 had a similar effect, whereas lower concentrations had no obvious effects. At a concentration of 25 µmo/L, potential PhoQ inhibitor 2 reduced cell invasion of the bacterium to 3.6×10^−1^, 16.3 times lower than the control, concentrations lower than 25 µmo/L had no obvious effects. Potential PhoQ inhibitor 3, at 100 µmo/L, reduced invasion of HeLa cells by *Sf*9380 to 1.95×10^−2^, concentrations lower than 100 µmo/L had no obvious effects. Potential PhoQ inhibitor 4, at 100 µmo/L, had no effect on the bacterial invasion of HeLa cells (shown in Figure 6A). For intracellular growth and spreading to adjacent cell, potential PhoQ inhibitor 1 (12.5 µmo/L) reduced cell invasion of bacterium to 0.061, 1.96 times lower than that of untreated *Sf*9380 (0.12), potential PhoQ inhibitor 2 (25 µmo/L) was reduced to 0.036, 3.3 times lower than that of untreated *Sf*9380, PhoQ inhibitor 3 (100 µmo/L) was reduced to 0.046, 2.6 times lower than that of untreated *Sf*9380, while PhoQ inhibitor 4 (100 µmo/L) have no obvious effect on the cell intracellular growth (0.12) (shown in Figure 6B).

The gentamicin protection assay shows the inhibitory effect of the four potential PhoQ inhibitors on *S. flexneri 2a* 301 *phoQ/phoP* knock-out mutant (*Sf*301 *ΔphoQ/phoP*) HeLa cells invasion. The results of the HeLa cell invasion showed that the cell invasion rate of *Sf*301 *ΔphoQ/phoP* treated with potential PhoQ inhibitor 1 (12.5 µmo/L), 2 (25 µmo/L), 3 (100 µmo/L), 4 (100 µmo/L) were 0.064, 0.102, 0.083 and 0.053, respectively. The cell invasion rate of *Sf*301 *ΔphoQ/phoP* was 0.069 (shown in Figure 6C).

**Figure 7:**
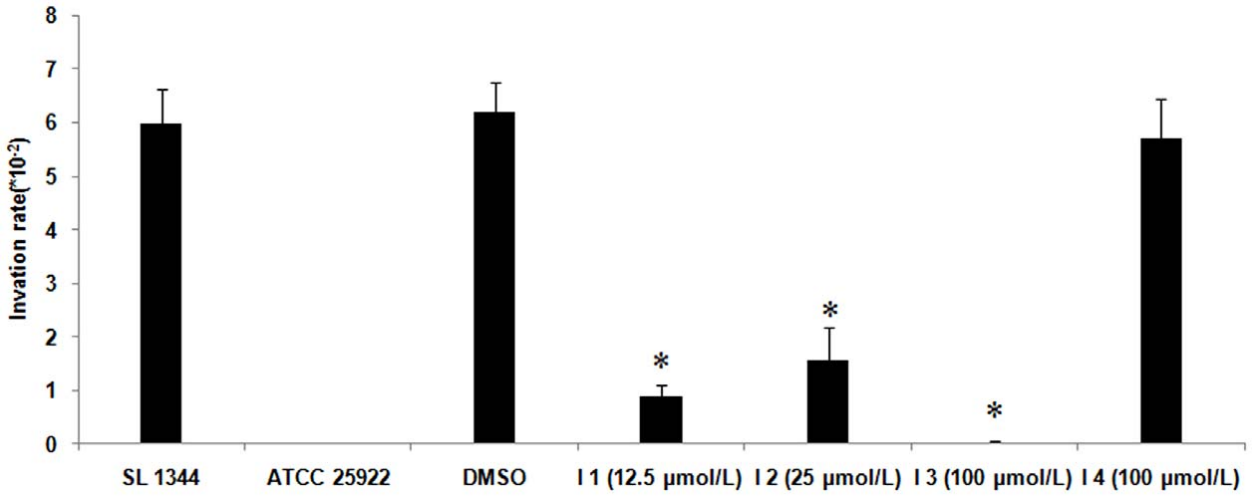
The effects of potential PhoQ inhibitors on cell invasion of *Salmonella*. The gentamicin protection assay shows the inhibitory effect of the four potential PhoQ inhibitors on *Salmonella enterica typhimurium SL*1344 HeLa cells invasion. The bacteria pre-treated with potential PhoQ inhibitor were added to HeLa cells at a multiplicity of infection of 100. Then gentamicin was added to kill extracellular bacteria and cell lysates were plated onto LB plates, and the colonies were counted. The results of the assays were expressed as the number of bacteria recovered from gentamicin-treated cells divided by the number of inoculated bacteria added to the cell. *Salmonella enterica typhimurium SL*1344 served as the positive control for the cell invasion assay; *E. coli* ATCC 25922 (treated without DMSO), an avirulent strain, served as the negative control for the cell invasion assay; bacteria treated with 0.1% DMSO also served as a control for the cell invasion assay. Values are means ± standard deviations from 6 independent wells. *p<0.01 vs. *SL*1344.

The effects of potential PhoQ inhibitors on cell invasion of *Salmonella* were determined by gentamicin protection assay. It showed that the cell invasion rate of *Salmonella enterica Typhimurium SL*1344 treated with potential PhoQ inhibitor 1 (12.5 µmo/L), 2 (25 µmo/L), 3 (100 µmo/L) were 0.89×10^−2^ (6.74 times lower than the control), 1.564×10^−2^ (3.84 times lower than the control) and 0.036×10^−2^ (166 times lower than the control), respectively, while potential PhoQ inhibitor 4 (100 µmo/L) had no obvious effect on the cell invasion (the cell invasion rate was 5.7×10^−2^). The cell invasion rate of *Salmonella* was 6×10^−2^ (shown in Figure 7).

### Effects of the potential PhoQ inhibitors on *S. flexneri* virulence *in vivo*

**Figure 8:**
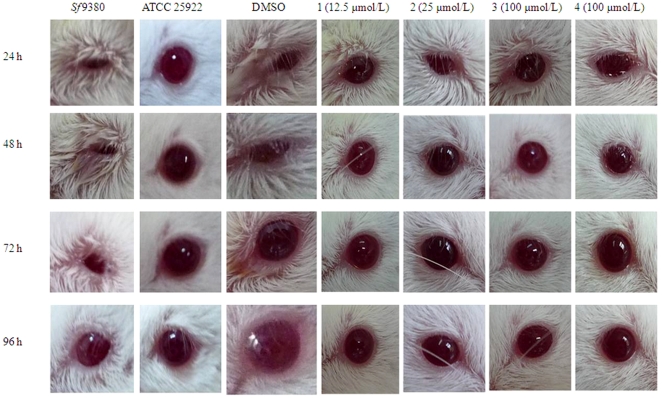
Potential PhoQ inhibitors can reduce the degree of keratoconjunctivitis produced by *Shigella* in mice. A Mouse Sereny test shows the virulence of *S. flexneri* with or without potential PhoQ inhibitor treatment. Mice inoculated with 1×10^8^ CFUs of *S. flexneri Sf*9380 show severe keratoconjunctivitis without purulence at 24 hours after infection, which develops to keratoconjunctivitis with purulence after 48 hours and continues for 96 hours. *Sf*9380 cultured with a serial dilution of potential PhoQ inhibitors were also inoculated into mouse eyes at 1×10^8^ CFUs. Mice inoculated with the bacteria pre-treated with potential PhoQ inhibitors 1 (12.5 µmol/L) or potential PhoQ inhibitors 2 (25 µmol/L) display a slight conjunctival inflammation at 24 hours post-infection, and keratoconjunctivitis is resolved by 48 hours. Mice inoculated with the bacteria pre-treated with potential PhoQ inhibitors 3 (100 µmol/L) or potential PhoQ inhibitors 4 (100 µmol/L) display a slight conjunctival inflammation at 24 hours post-infection, and keratoconjunctivitis is resolved by 72 hours. Mice inoculated with the negative control, *E. coli* ATCC 25922, did not develop conjunctival inflammation. *Sf*9380 treated with the same amount of DMSO in which the potential PhoQ inhibitors were dissolved served as a control.

A Mouse Sereny test was used to investigate the inhibitory effect of the four potential PhoQ inhibitors on the virulence of *S. flexneri Sf*9380. The mice were infected with 1×10^8^ CFUs per eye. Mice inoculated with *Sf*9380 showed severe keratoconjunctivitis without purulence at 24 hours post-infection, which developed to keratoconjunctivitis with purulence after 48 hours, and the condition was sustained for 96 hours (Figure 8, Table 2). Mice inoculated with bacteria pre-treated with potential PhoQ inhibitors 1 (12.5 µmol/L) or potential PhoQ inhibitors 2 (25 µmol/L) displayed a slight conjunctival inflammation at 24 hours after infection, but keratoconjunctivitis was resolved by 48 hours (Figure 8, Table 2). Mice inoculated with bacteria pre-treated with potential PhoQ inhibitor 3 (100 µmol/L) or potential PhoQ inhibitor 4 (100 µmol/L) displayed a slight conjunctival inflammation 24 hours post-infection, with keratoconjunctivitis resolved by 72 hours (Figure 8, Table 2). Mice infected with *S. flexneri Sf*9380 treated, with the same amount of DMSO (0.1%, v/v) as the potential PhoQ inhibitors, displayed severe keratoconjunctivitis (Figure 8, Table 2). Mice inoculated with *E. coli* ATCC 25922 did not develop conjunctival inflammation as a negative control (Figure 8, Table 2).

**Table 2:** Keratoconjunctivitis in mice inoculated with potential PhoQ inhibitors treated *Sf*9380.

	24 h						48 h						72 h						96 h					
	1	2	3	4	5	6	1	2	3	4	5	6	1	2	3	4	5	6	1	2	3	4	5	6
*Sf*9380(a)	++	++	+	++	++	++	+++	++	+++	++	++	+++	++	++	+++	++	++	++	++	+	++	++	+	+
ATCC 25922(b)	−	−	−	−	−	−	−	−	−	−	−	−	−	−	−	−	−	−	−	−	−	−	−	−
DMSO(c)	++	++	+	++	++	++	+++	+++	++	+++	++	++	++	++	++	++	++	++	++	+	++	+	++	+
I 1 (12.5 µmol/L)	+	+	+	−	+	+	−	−	+	−	−	−	−	−	−	−	−	−	−	−	−	−	−	−
I 2 (25 µmol/L)	+	+	+	+	+	+	−	+	−	−	−	−	−	−	−	−	−	−	−	−	−	−	−	−
I 3 (100 µmol/L)	+	+	+	+	+	+	+	−	−	−	+	−	−	+	−	−	−	−	−	−	−	−	−	−
I 4 (100 µmol/L)	+	+	+	+	+	−	+	−	−	−	+	−	+	−	−	−	−	−	−	−	−	−	−	−

A Mouse Sereny test shows the virulence of *S. flexneri Sf*9380. Mice inoculated with 1×10^8^ CFUs display severe keratoconjunctivitis without purulence 24 hours post-infection, developed keratoconjunctivitis with purulence after 48 hours, and sustained the infection for 96 hours. *Sf*9380 cultured with a serial dilution of potential PhoQ inhibitors was inoculated into mice, at 1×10^8^ CFUs per eye.

The degree of keratoconjunctival inflammation in each of the six mice infected with *Sf*9380, ATCC 25922, DMSO, and *Sf*9380 pre-treated with different concentrations of potential PhoQ inhibitors 1, 2, 3 or 4 at 24, 48,72 and 96 hours (n = 6). Mouse Keratoconjunctivitis was rated as follows: −, no disease; ±, little keratoconjunctivitis; +, mild keratoconjunctivitis; ++, keratoconjunctivitis with some purulence; +++, fully developed keratoconjunctivitis with great purulence.

(a) Inoculation of *Sf*9380 alone, served as the positive control for the Mouse Sereny.

(b) ATCC 25922, an avirulent *E. coli* strain, served as a negative control for the Mouse Sereny test.

(c) DMSO, *Sf*9380 treated with DMSO, the solvent of the potential PhoQ inhibitors, served as a control for the Mouse Sereny test.

### Cytotoxicity assay and erythrocyte hemolysis assays of potential PhoQ inhibitors

The cytotoxicity of each potential PhoQ inhibitor towards a Vero cell line was analyzed by the traditional MTT assay. The CC_50_ values of all four compounds were higher than 200 µmol/L, the highest tested concentration of potential PhoQ inhibitors. As a control, an equal concentration of DMSO (0.1%) was added to a well of Vero cells, and no obvious cytotoxicity was observed (Table 1).

To determine whether the four potential PhoQ inhibitors would induce hemolysis of mammalian erythrocytes, the hemolytic activities of each potential PhoQ inhibitor were tested at concentrations of 25 µmol/L and 100 µmol/L. All four potential PhoQ inhibitors showed no hemolytic activity (Table 1).

## Discussion

Currently, there is an increase in antibiotic resistance among *Shigella* isolates [1], and this drug resistance phenomenon is causing complications and difficulties for clinical treatment. Several virulence regulator factors, such as two-component signal systems [10], [11], [12], quorum sensing systems [11], [13], [14], type III secretion systems [15], and the assembly of adhesive organelles [16], have been recognized as interesting targets to reduce bacterial infection. Bacterial two-component systems have gained increasing interest as novel antibacterial targets because these systems are required for virulence of pathogenic microorganisms [17], [18]. In the present study, we found that the PhoQ/PhoP two-component system of *Shigella* may be a promising target for developing new antibiotics against *S. flexneri* infection.

PhoQ/PhoP is a two-component system that governs virulence [4], monitors the extracellular Mg^2+^, and regulates several cellular activities in many gram-negative species. The system also helps bacteria resist antibiotic peptides by regulating lipid A [7], [19], [20], [21], [22]. Bivalent cations and antibiotic peptides can competitively bind to the acidic structural domain on the cytoplasmic surface of PhoQ [23]. In addition to the concentration of Mg^2+^ or Ca^2+^cations in the cytoplasm, it has been shown that the concentration of antibiotic peptides in the external environment [24], in addition to an acidic environment, will mediate the activation of PhoQ [25]. In *Salmonella*, PhoQ/PhoP can change the structure of the external cell membrane by regulating the remodeling of lipid A to strengthen a bacterium's resistance to the environment [26]. In *Shigella*, the PhoQ/PhoP two-component system is necessary for virulence, as demonstrated by an infection of mice with a *phoP* mutant of *Shigella* that resulted in milder keratoconjunctivitis than a wild type strain [7].

PhoQ is an attractive target for an antibiotic because it is absent in mammals [10], [27]. In this study, we have explored the possibility of using the PhoQ as a potential target by performing a screen for inhibitors. After constructing a 3D model of the PhoQ HK domain of *Sf*301, 64 compounds were selected as inhibitor candidates based on their molecular diversity, shape complementarities, and potential for forming hydrogen bonds in the binding pocket of PhoQ. To confirm the interaction of the compounds and PhoQ, a prokaryotic expression plasmid containing the *Sf*301 PhoQ intracellular domain which contains HK domain was constructed, because the main biology activity of PhoQ is depends on its HK domain [28]. To confirm whether these inhibitor candidates targeted the PhoQ HK domain, enzymatic activities of PhoQ were determined in the presence or absence of four compounds. The enzymatic activity of SF-PhoQc was measured using both a Pyrophosphate Reagent and a Luminescent Kinase Assay. The Pyrophosphate Reagent can reflect the reaction of HK and ATP at real time, but not sensitive. The Luminescent Kinase Assay is more sensitive than Pyrophosphate Reagent for kinase reaction but cannot reflect the reaction of HK and ATP at real time. Therefore, in the present study we used two assays to confirm the results. The different IC_50_ values of potential PhoQ inhibitors 1 and 3 determined by the two assays may be the sensitivity difference between the two assays.

By using cell invasion assays, the features of cell invasion process including penetration into epithelial cells and spreading to adjacent cells were tested [29]. The *Shigella* (*Sf*9380) were treated with four potential PhoQ inhibitors (100 µmol/L) for 4, 6 or 8 hours, respectively. Compared with cell invasion of the positive control *Sf*9380 alone, the potential PhoQ inhibitors (100 µmol/L) treated for 8 hours had obvious inhibition effects on the bacteria cell invasion by using gentamicin protection assay, while potential PhoQ inhibitors treated for 4 or 6 hours had no significant inhibition effects on *Sf*9380 cell invasion (Figure S3). Therefore, *Shigella* cell invasion assay and Mouse Sereny test were carried out by the bacteria treated with potential PhoQ inhibitors for 8 hours.

To confirm these four potential PhoQ inhibitors were affecting PhoQ histidine kinase, we made a *S. flexneri phoQ/phoP* knock-out mutant (*Sf*301 *ΔphoQ/phoP*) and the cell invasion ability was tested. The results indicated that potential PhoQ inhibitors 1, 2, 3 can inhibit HeLa cell invasion ability of *Sf*301 (Figure S4) but have no obvious effects on *Sf*301 *phoQ/phoP* knock-out mutant (Figure 6C). It indicated that these potential PhoQ inhibitors can affect PhoQ histidine. The results also suggested that the cell invasion ability of *Sf*301 *ΔphoQ/phoP* decreased significantly compared to the wild type strain. It indicated that *phoQ/phoP* could regulate the cell invasion of *S. flexneri*.

The PhoQ of *Salmonella* is high homology to that of *Shigella* and in *Salmonella* PhoP/PhoQ regulates virulence including cell invasion [4], [6]. So it was curious for us to evaluate whether these four potential PhoQ inhibitors have similar effect on *Salmonella*. The results of cell invasion suggested that cell invasion of *Salmonella SL*1344 was inhibited by the potential PhoQ inhibitors 1, 2, 3 while potential PhoQ inhibitor 4 has no obvious effect on cell invasion which were similar to the results of *Shigella* (Figure 7). It indicates that the potential PhoQ inhibitors can inhibit the cell invasion of *Salmonella*.

In the present study, we chose the PhoQ protein, a transmembrane sensor of the PhoQ/PhoP TCS in *S. flexneri 2a* 301, as the target and found that three potential PhoQ inhibitors can inhibit the bacterial ability to invade HeLa cells. Further, we found out that with *phoQ/phoP* knocking out, the cell invasion ability of *Sf*301 *ΔphoQ/phoP* decreased significantly, compared to the wild type strain, and no obvious effects of potential PhoQ inhibitors on *Sf*301 *ΔphoQ/phoP* were observed. However, Moss et al has reported that there were no significant differences between wild-type and *phoP* mutant of *S. flexneri serotype 5 strain* M90T in HeLa cell invasion [7]. In gram-negative pathogen, there are many cross-talks between two-component systems (TCSs) [30], [31], [32], [33], in which one HK (Histidine Kinase) can regulate several RRs (Response Regulator), and one RR's phosphorylation can be regulated by several HKs to gather several signal pathways of TCSs and induce expression of a battery of downstream genes. The difference between *phoQ/phoP* knock-out mutant of *S. flexneri 2a* 301 and *phoP* knock-out mutant of *S. flexneri 5* M90T may due to the cross-talks between TCSs in the bacterium, although in *Shigella* it remains poorly understood. Involvement of the PhoQ/PhoP cascade on *Shigella* virulence across strains, serotypes and species need to be investigated in the future.

In the present study, four potential PhoQ inhibitors, at 200 µmol/L, showed no effect on *Shigella* growth (Figure S2). This was expected because the PhoQ/PhoP signaling system does not directly regulate bacterial growth. With increasing knowledge about bacterial virulence, several researchers have found that bacterial virulence genes are essential to mount a harmful infection, but they are usually dispensable for growth of bacteria *in vitro*. These results indicate that inhibition of microbial virulence without inhibiting their growth may be a promising strategy [34]. In contrast, currently available antibiotics either kill bacteria or prevent their growth. Drugs that block disease without killing the pathogen bacteria [35], [36] may cause less selective pressure for the generation of drug resistance [34], [36], [37]. These alternative drug strategies would presumably induce pathogen resistance at a much slower rate because the targeted non-essential genes or functions are under less selective pressure to mutate. The host will be subjected to intact avirulent bacteria, allowing the host to develop an adequate immune response against the pathogen. This would allow the host to efficiently respond to and eradicate an invader upon re-exposure. Therefore, the strategy to target bacterial virulence factors has become an attractive approach for the development of new therapeutic agents [35].

In addition to novel drug targets, the use of small organic molecules is gaining interest more than genetic-based drugs [38], [39]. Small organic molecules that target specific proteins may be used for the prevention or treatment of infections caused by a wide variety of gram-negative bacteria species, including *Escherichia coli* [17], [40], *Salmonella typhimurium* [18] and *Yersinia pseudotuberculosis* [41], as well as gram-positive bacteria such as *Staphylococcus epidermidis* [42]. In this study, four promising potential PhoQ inhibitor candidates were validated using enzymatic activity assays and binding affinities.

In previous studies, some potential PhoQ inhibitors displayed side effects, such as membrane damage or excessive protein binding, which would be an obstacle for their further development [43]. In this study, we found four potential PhoQ inhibitors that reduce the virulence of *Shigella* that also have low cytotoxicity and hemolysis of mammalian cells at their effective concentrations. We demonstrated that PhoQ/PhoP is a promising target for the development of new drugs against *S. flexneri* infection and proved that four potential PhoQ inhibitors can inhibit the virulence of *Shigella*. In future work, we will modify the compound structure to increase the efficacy of the potential PhoQ inhibitors and identify which stage of infection is inhibited by these potential inhibitors which is important to the therapy of shigellosis.

## Materials and Methods

### Ethics Statement

All procedures performed on mice were conducted according to relevant national and international guidelines (the Regulations for the Administration of Affairs Concerning Experimental Animals, China, and the NIH Guide for the Care and Use of Laboratory Animals) and were approved by the Institutional Animal Care and Use Committee (IACUC) of Shanghai Medical College, Fudan University (IACUC Animal Project Number: 20090601-qu).

### Bacterial strains, growth conditions and reagents

*S. flexneri 2a* 301 (*Sf*301, Genbank Accession number for chromosomes is AE005674) was kindly provided by Pr. Jianguo Xu (Chinese Center for Disease Control and Prevention, Beijing, China). A clinical strain of *S. flexneri*, *Sf*9380, was isolated from an epidemic in the Hebei province by Dr. Xuanyi Wang from our laboratory. *E. coli* ATCC 25922 was kindly provided by Dr. Bijie Hu (Zhongshan Hospital, Shanghai, China). *S. enterica typhimurium SL*1344 and plasmid pSB890 were kindly provided by Dr Daoguo Zhou (Purdue University, USA). *E. coli* JM109 and plasmid pQE30 were purchased from Qiagen (Germany). *E. coli* JM109 was used for both recombinant DNA manipulation and protein production. *S. flexneri* and *E. coli* were grown at 37°C in Luria–Bertani medium (LB; Oxoid, Wesel, Germany). All compounds used as inhibitor candidates were purchased from the SPECS Company (Netherlands). Stock solutions (10 mmol/L) of the potential PhoQ inhibitors were prepared in dimethyl sulfoxide (DMSO).

### 3D structure modeling of the PhoQ histidine kinase of *S. flexneri Sf*301

The PhoQ histidine kinase sequence of *S. flexneri 2a* strain 301 was retrieved from GenBank (accession number AE005674). To identify potential inhibitors of PhoQ histidine kinase (PhoQ HK) by virtual screening, we obtained the crystal structure of the PhoQ HK domain (PDB entry code: 1ID0) from the Brookhaven Protein Database (PDB http://www.rcsb.org/pdb). The missing residues in the PhoQ HK were added and subjected to energy minimization in Sybyl 6.8 [43] using the steepest descent method up to the gradient tolerance of 0.05 kcal/(mol·Å) to relieve possible steric clashes and overlaps of side chains. The potential of the 3D structure of PhoQ HK domain was assigned according to the Amber force field with Kollman-united-atom charges encoded in Sybyl 6.8 [43].

### Structure-based virtual screening of potential PhoQ HK inhibitors

The ATP-binding pocket formed by residues within a radius of 7 Å around the ATP site of the PhoQ HK catalytic domain was used as the target site for high-throughput virtual screening (HTVS). The optimized 3D model of PhoQ HK was used as the target for virtual screening with the SPECS database (http://www.specs.net). In the first step, 85,000 potential drug-like molecules were selected from the SPECS database, creating an in-house database (named SPECS_1). Next, the SPECS_1 database was searched for potential binding molecule structures using the program DOCK4.0 [44], [45]. The most optimal 10,000 structures were subsequently re-scored using the FlexX [46] and CSCORE, a consensus scoring method that integrates five popular scoring functions. Two hundred molecules passed this highly selective filter. Finally, 100 molecules were manually selected as inhibitor candidates, according to their molecular diversity, their shape complementarities, and their potential for forming hydrogen bonds in the binding pocket of the PhoQ HK domain.

### Expression and purification of recombinant SF-PhoQc

A DNA segment encoding the cytoplasmic domain of PhoQ HK was amplified by PCR from the *Sf*301 genomic DNA with primers PhoQc5′ (5′-CGCGGATCCGTACGAAACCTGAACCGATT-3′) and PhoQc3′ (5′-CCCAAGCTTTTATTCATCTTTCGGCGCAG-3′), which have BamHI and HindIII restriction sites, respectively. The amplified DNA segment encodes PhoQ HK amino acids 251–486, which include the phosphoester and catalytic domains of PhoQ HK (SF-PhoQc). The amplified target DNA was purified, digested by BamHI and HindIII (TaKaRa Biotechnology Co., Ltd, China) and cloned into pQE30, creating pQE30-SF-PhoQc. The prokaryotic expression plasmid pQE30-SF-PhoQc was constructed to produce a PhoQc and N-terminal hexahistidine fusion protein. The pQE30-SF-PhoQc plasmid was transformed into *E. coli* JM109 and cultured overnight at 37°C in LB containing 100 µg/ml of ampicillin. When the bacteria reached OD600 absorbance values of 0.5–0.7, expression of SF-PhoQc was induced by adding 0.8 mmol/L isopropyl-β-D-thiogalacto-pyranoside (IPTG). Incubation continued under rigorous shaking at 30°C for 6 hours. Cells were harvested at 10,000×g for 30 minutes, resuspended in buffer-A (20 mmol/L Tris, pH 8.0, 300 mmol/L NaCl and 10 mmol/L imidazole), and disrupted by sonication on ice. The lysed bacteria were centrifuged to yield a clear supernatant, and the supernatant was loaded onto a column with Ni-NTA resin (Qiagen) pre-equilibrated in buffer A. The column was washed with buffer B (20 mmol/L Tris, pH 8.0, 300 mmol/L NaCl, 30 mmol/L imidazole) and eluted with buffer C (20 mmol/L Tris, pH 8.0, 300 mmol/L NaCl, 200 mmol/L imidazole). The eluted fractions were concentrated and dialyzed against buffer D (20 mmol/L Tris, pH 8.0, 150 mmol/L NaCl) to remove imidazole and stored at −70°C. The purified protein was identified by AutoFlex MALDI-MS (Bruker Daltonics, Inc., Billerica, MA, USA). The operating condition was optimized with standard solution, and the working parameters of the ion source were as follows: lasing light emitter N2, wave length 337 nm; cation with accelerating voltage 20 kV and matrix SA.

### Enzymatic activity assays of SF-PhoQc with the potential PhoQ inhibitors

The enzymatic activity of SF-PhoQc was measured using both a Pyrophosphate Reagent (Sigma-Aldrich, MO, USA) and a Luminescent Kinase Assay (Promega, WI, USA). The Pyrophosphate Reagent was performed to confirm the inhibitory activities of the effective potential PhoQ inhibitors in the bioinformatics approach. All reactions were carried out in solid black, flat-bottomed 96-well plates. The Pyrophosphate Reagent was performed at 28°C by monitoring the decrease of NADH by a decrease in absorbance readings at 340 nm (ε340 = 6180 M^−1^·cm^−1^) in the presence of ATP. The final reaction mixture for the Pyrophosphate Reagent contained 25 µmol/L purified SF-PhoQc, 25 mmol/L KCl, 2.5 mmol/L MgCl_2_, 2 mmol/L ATP, 1 mmol/L phosphoendpyruvate, 0.35 mmol/L NADH, 1.5 U/ml PK, 1.25 U/ml LDH and 50 mmol/L Tris-Cl (pH 8.0). The enzyme activity was determined in the presence of various concentrations of potential PhoQ inhibitors (0–100 mmol/L) to investigate dose-dependent inhibition effects. The A_340_ value was recorded by SoftMax vision 4.8 every minute, for 30 minutes. The rate of SF-PhoQc enzymatic activity inhibition by a compound was calculated by the following formula:
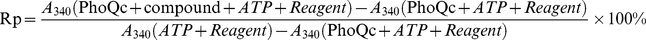



Rp (Ratio of Phosphorylation) was defined as the ratio of the decrease activity of SF-PhoQc treated with compound vs the activity of SF-PhoQc treated without compounds. Rp was stand for the percentage of decrease activity of SF-PhoQc.

IC_50_(the concentration of inhibition of 50%)values of potential PhoQ inhibitors were obtained by fitting the data to a sigmoid dose–response equation using Origin 7.0 software (OriginLab, Northampton, USA).

In the Luminescent Kinase assay, reactions were performed in solid black, flat-bottomed 96-well plates. To determine the IC_50_ of potential PhoQ HK inhibitors, each compound underwent 2-fold serial dilutions with DMSO (0.1%) and was added into the reaction mixture. The final reaction mixture contained 4 µg of purified SF-PhoQc, 40 mmol/L Tris-Cl (pH 7.5), 16 mmol/L MgCl_2_, 0.8 mmol/L DTT and different concentration of 100, 50, 25, 12.5, 6.25 or 3.125 µmol/L compound, respectively. After incubation at 30°C for 25 minutes, 3 µmol/L ATP was added, and the reaction was incubated at 30°C for another 25 minutes. Next, an equal volume of Luminescent Kinase Reagent was added to each well, mixed and kept at room temperature for 10 minutes before recording the luminescence reading on a Fluoroskan Ascent FL machine (Thermo Scientific, USA). The rate of phosphorylation inhibition by a compound was calculated with the following formula:
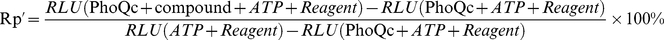



The IC_50_ value of each compound was determined using the Origin 7.0 software (OriginLab, Northampton, USA).

### Binding affinities of potential PhoQ inhibitors to SF-PhoQc

The binding affinities of potential PhoQ inhibitors to SF-PhoQc were determined *in vitro* using Surface Plasmon Resonance (SPR) technology with the dual flow cell Biacore 3000 instrument (Biacore AB, Uppsala, Sweden). The standard primary amine coupling reaction wizard immobilized SF-PhoQc to the hydrophilic carboxymethylated dextran matrix of the sensor chip, CM5 (Biacore). The SF-PhoQc covalently bound to the matrix was diluted in 10 mmol/L sodium acetate buffer (pH 4.2) to a final concentration of 0.035 mg/ml. Equilibration of the baseline was completed by a continuous flow of HBS-EP running buffer (10 mmol/L HEPES, 150 mmol/L NaCl, 3 mmol/L EDTA, and 0.005% (v/v) surfactant P20, pH 7.4) through the chip for 1–2 hours. All the Biacore data were collected at 25°C with HBS-EP as the running buffer at a constant flow of 30 µl/min. All the sensor grams were processed by using automatic correction for nonspecific bulk refractive index effects. The equilibrium constants (K_D_ values) evaluating the protein-ligand binding affinities were determined by the steady-state affinity fitting analysis of the Biacore data.

### Construction of the *S. flexneri phoQ/phoP* deletion mutant

The plasmid for *phoQ/phoP* deletion in *S. flexneri 2a* 301 was constructed. The upstream and downstream of the *phoQ/phoP* were amplified by using PCR with the primers listed in the Table 3: *phoQ/phoP* U-F and *phoQ/phoP* U-R were used to amplify upstream fragment; *phoQ/phoP* D-F and *phoQ/phoP* D-R were used to amplify downstream fragment. The amplified upstream fragment of *phoQ/phoP* was digested with NotI and XbaI restriction endonucleases, ligated into NotI and XbaI-cut pSB890 vector (a suicide vector [47]), resulting in a plasmid name as pSB890up. The amplified downstream fragment of *phoQ/phoP* was digested with XbaI and SmaI restriction endonucleases and ligated into XbaI and SmaI-cut pSB890up vector, resulting in a plasmid pSB890 *phoQ/phoP* with the upstream and downstream fragments of *phoQ/phoP*. The plasmid pSB890 *phoQ/phoP* was transferred into *E. coli* SM10λpir cells, then pSB890 *phoQ/phoP* was introduced into *S. flexneri 2a* 301 by conjugation. Equal volumes (5 ml) of *S. flexneri 2a* 301 wild-type recipient and *E .coli* SM10λpir contain pSB890 *phoQ/phoP* were mixed, and 100 µl was spotted onto an LB agar plate. After incubation (37°C for 24 h), the bacteria were recovered, resuspended in 1 ml of Luria-Bertani (LB) broth, and cultured on LB agar plate containing ampicillin and streptomycin at 37°C. Individual ampicillin-resistant *S. flexneri* colonies were inoculated into LB broth, grown overnight at 22°C, and plated onto LB agar supplemented with 5% sucrose. Sucrose-tolerant colonies were screened using PCR with primers 5′-CCCCGCTGGTTTATTTAATGTTTA-3′ and 5′-ACCCGCCTGTATATTTCTTGTGTC-3′. A *Sf*301 *ΔphoQ/phoP* was screened out and identified by PCR and sequencing.

**Table 3:** Primers used in homologous recombinations to construct knockout strain.

Primers	Sequences	Length (bp)	Restriction sites
*phoQ/phoP* U-F	5′ ATAAGAAT GCGGCCGC GCGTGACCTGACCGACTCCA 3′	528	NotI
*phoQ/phoP* U-R	5′ TGC TCTAGA TACGCGCATTTTTATTTCTCCCTGT 3′		XbaI
*phoQ/phoP* D-F	5′ TGC TCTAGA GATGAATAAATATGTCCTTTTACC 3′	555	XbaI
*phoQ/phoP* D-R	5′ TCC CCCGGG CCTCAATCGTGACGGAGA 3′		SmaI

The underlined sequences represent the restriction sites. Primers *phoQ/phoP* U-F and *phoQ/phoP* U-R were used to amplify upstream fragment of *phoQ/phoP*; *phoQ/phoP* D-F and *phoQ/phoP* D-R were used to amplify downstream fragment of *phoQ/phoP*.

### *S. flexneri* cell invasion assay

Bacterial ability to invade HeLa cells was tested with a gentamicin protection assay [9]. HeLa cells were grown in six-well tissue culture plates to semi-confluent monolayers (DMEM, 10% FCS, 5% CO_2_). *Sf*9380 or *Sf*301 *ΔphoQ/phoP* was treated with potential PhoQ inhibitors for 8 hours in LB at 37°C (final concentrations of 100,50,25,12.5 or 6.25 µmol/L) prior to infection. The bacteria pre-treated with potential PhoQ inhibitor without washing, were added to semi-confluent HeLa cells at a multiplicity of infection of 100, at the same time the bacteria were diluted and plated onto LB agar plates. The CFUs were counted as initial bacterial number before added into HeLa cells. Then the plates were centrifuged at 900×g for 5 minutes. After incubating at 37°C for 60 minutes (For testing intracellular growth and spreading to adjacent cell, cells were further incubated for 290 min [9].), the cells were washed three times with PBS, and gentamicin was added to the medium to a final concentration of 10 µg/ml for 20 minutes at 37°C. After the incubation, the HeLa cells in each well were lysed in 1 ml of PBS containing 0.1% Triton X-100 for 10 minutes at room temperature. The lysates were diluted and plated onto LB agar plates in triplicate. Colonies grown on LB plates were counted. The results of the assays were expressed as the number of bacteria recovered from gentamicin-treated cells divided by the number of inoculated bacteria added to the cell. The cells were inoculated with untreated *Sf*9380 or *Sf*301 *ΔphoQ/phoP* (treated without DMSO) as a positive control and *E. coli* ATCC 25922, an avirulent strain treated without DMSO, as a negative control. DMSO is the solvent of the potential PhoQ inhibitor compounds; therefore, *Sf*9380 was also treated with the same amount of 0.1% DMSO as the other groups to control for any DMSO-specific effects. The cell invasion assay was performed in triplicate for each potential PhoQ inhibitor, and the assay was repeated twice.

### *Salmonella* cell invasion assay

The gentamicin protection assay was used as a cellular model to evaluate the inhibitory effect of the four potential PhoQ inhibitors. *Salmonella enterica typhimurium SL*1344 was treated with each of the four potential PhoQ inhibitors (final concentrations: potential PhoQ inhibitor 1 was 12.5 µmol/L, 2 was 25 µmol/L, 3 was 100 µmol/L and 4 was 100 µmol/L) and grown for 8 hours. The bacteria pre-treated with potential PhoQ inhibitor without washing, were added to HeLa cells at a multiplicity of infection of 100, at the same time the bacteria were diluted and plated onto LB agar plates. The CFUs were counted as initial bacterial number before added into HeLa cells. Next, the bacteria were inoculated to HeLa cells and incubated with for 60 minutes prior to the addition of gentamicin to kill extracellular bacteria. After the incubation, the HeLa cells in each well were lysed in 1 ml of PBS containing 0.1% Triton X-100 for 10 minutes at room temperature. The lysates were diluted and plated onto LB agar plates in triplicate. Colonies grown on LB plates were counted. The results of the assays were expressed as the number of bacteria recovered from gentamicin-treated cells divided by the number of inoculated bacteria added to the cell. After lysates of HeLa cells were plated, the number of CFU that formed on LB agar were counted. The cells were inoculated with untreated *SL*1344 (treated without DMSO) as a positive control and *E. coli* ATCC 25922, an avirulent strain treated without DMSO, as a negative control. *SL*1344 was treated with the same amount of 0.1% DMSO as the other groups to control for any DMSO-specific effects. The cell invasion assay was performed in triplicate for each potential PhoQ inhibitor, and the assay was repeated twice.

### *S. flexneri* Mouse Sereny test

A Mouse Sereny test was used to evaluate the virulence of *S. flexneri Sf*9380 [48]. A single red colony of *Sf*9380 on Congo Red agar (Tryptic soy broth provided by Oxfoid Co. with 1.5% agar and 0.01% Congo red) [48], was inoculated into LB and grown with or without potential PhoQ inhibitors (final concentration 100,50,25,12.5 or 6.25 µmol/L) at 37°C for 8 hours with constant shaking. The female BALB/c mice (about 18 g) were infected with 1×10^8^ CFUs per eye. The eye of the mouse was inoculated with the bacterium with or without potential PhoQ inhibitor treatment (n = 6 mice in each group). Keratoconjunctivitis in the mice infected with bacteria was observed at 24, 48, 72 and 96 hours after inoculation. In the experiment, mouse eyes inoculated with untreated *Sf*9380 (treated without DMSO) served as the positive control for keratoconjunctivitis, and mouse eyes inoculated with *E. coli* ATCC 25922 (treated without DMSO) served as the negative control. Because DMSO is the solvent of the potential PhoQ inhibitor, *Sf*9380 was treated with the same amount of DMSO (0.1%) as the other groups. The invasiveness of the bacteria was scored: no disease or mild irritation as “−”, + as mild conjunctivitis or late development and/or rapid clearing of symptoms, ++ as keratoconjunctivitis without purulence, and +++ as fully developed keratoconjunctivitis with purulence [49].

### Cytotoxicity assay of the potential PhoQ HK inhibitors

Cytotoxicity of the potential PhoQ inhibitors on cultured Vero cells was measured by using the Cell Proliferation Kit I (MTT) (Roche, Indianapolis, USA). An equal concentration of DMSO (0.1%) in the medium was used as a negative control. The cytotoxicity assay was performed in triplicate for each potential PhoQ inhibitor, and the assay for each potential PhoQ inhibitor was repeated twice. The results were converted to percentage of the control (cells only containing 0.1% DMSO) and CC_50_ (concentrations that produce a 50% cytotoxicity effect on Vero cell) of each potential PhoQ inhibitors was calculated using the Origin v7.0 software (OriginLab, Northampton, USA).

### Erythrocyte hemolysis assays of potential PhoQ inhibitors

The hemolytic activity of the potential PhoQ inhibitors was determined using human erythrocytes [50]. The erythrocytes were washed three times with sterile saline and resuspended to 5% cell concentration prior to assay. Then, 200 µl erythrocyte suspensions containing 25 µmol/L or 100 µmol/L concentrations of each potential PhoQ inhibitors were added in triplicate to the wells of the 96-well microtiter plates. Cells without potential PhoQ inhibitors treatment or with 1% Triton-100 treatment were used as the zero or 100% hemolysis control, respectively. The cell suspensions were incubated for 1 hour at 37°C and centrifuged for 10 minutes at 1200×g. Supernatants (100 µl) were transferred to another sterile plate, and the released hemoglobin was measured absorbance readings at 570 nm. An equal concentration of DMSO (0.1%) in the medium did not affect the integrity of the erythrocyte membrane. The hemolysis assay was repeated twice.

## Supporting Information

Figure S1 **Identification of purified recombinant SF-PhoQc.** (**A**) Purified recombinant SF-PhoQc was analyzed by SDS-PAGE. The theoretical molecular mass calculated according to the amino acid sequence of SF-PhoQc is 27.9 kDa. (**B**) The purified recombinant protein was confirmed by Western blot with a monoclonal antibody against the His tag. (**C**) The molecular mass of the protein was determined by mass spectrometry. The MS spectral data shown gives a 27.914 kDa molecular mass of the recombinant SF-PhoQc, which matches the theoretical molecular mass of 27.914 kDa calculated from the amino acid sequence.
(TIF)

Figure S2 **The potential PhoQ inhibitors have no effects of on** ***Shigella*** **growth.** The growth curves of *Sf*9380 which treated with four potential PhoQ inhibitors were tested. *Sf*9380 inoculated with potential PhoQ inhibitors (final concentration 200 µmol/L), and Bacterial Concentration (OD 600) of *Sf*9380 in the culture were counted every one hour, for 12 hours. X-axis was the hours of bacterial growth, Y-axis was the Bacterial Concentration (OD 600). These result shows that four potential PhoQ inhibitors can not affect the growth of *S. flexneri*.
(TIF)

Figure S3 **Duration of treatment influences the effects of potential PhoQ inhibitors on cell invasion of** ***Shigella*** **.** The gentamicin protection assay shows the inhibitory effect of the four potential PhoQ inhibitors on *Shigella* HeLa cells invasion. The *S. flexneri Sf*9380 were treated with each of the four potential PhoQ inhibitors (100 µmo/L) grown for 4, 6 or 8 hours. Then the bacteria were inoculated with HeLa cells for 60 minutes prior to the addition of gentamicin to kill extracellular bacteria. After the incubation, the HeLa cells in each well were lysed in 1 ml of PBS containing 0.1% Triton X-100. The lysates were diluted and plated onto LB agar plates in triplicate. Colonies grown on LB plates were counted. The results of the assays were expressed as the number of bacteria recovered from gentamicin-treated cells divided by the number of inoculated bacteria added to the cell. Result indicated that compared with cell invasion of the positive control *Sf*9380 alone, the potential PhoQ inhibitors (100 µmol/L) treated for 8 hours had obvious inhibition effects on the bacteria cell invasion, while potential PhoQ inhibitors treated for 4 or 6 hours had no significant inhibition effects on *Shigella* cell invasion.
(TIF)

Figure S4 **The effects of potential PhoQ inhibitors on cell invasion of** ***Shigella Sf*** **301.** The gentamicin protection assay shows the inhibitory effect of the four potential PhoQ inhibitors on *S. flexneri 2a* 301 HeLa cells invasion. The bacteria were treated with each of the four potential PhoQ inhibitors grown for 8 h. Then the bacteria were inoculated with HeLa cells for 60 minutes prior to the addition of gentamicin to kill extracellular bacteria. After the incubation, the HeLa cells in each well were lysed in 1 ml of PBS containing 0.1% Triton X-100. The lysates were diluted and plated onto LB agar plates in triplicate. Colonies grown on LB plates were counted. The results of the assays were expressed as the number of bacteria recovered from gentamicin-treated cells divided by the number of inoculated bacteria added to the cell. Values are means ± standard deviations from 6 independent wells. *p<0.01 vs. *Sf*301. The results indicated that potential PhoQ inhibitors 1, 2, 3 can inhibit HeLa cell invasion ability of *Sf*301 while potential PhoQ inhibitor 4 had no effect on *Sf*301 invasion of HeLa cells.
(TIF)
