# Supplementary figures and images for: The Effect of the Potential PhoQ Histidine Kinase Inhibitors on Shigella flexneri Virulence
Source: PLoS One. 2011 Aug 10;6(8):e23100. doi: 10.1371/journal.pone.0023100 (PMC3154276; doi:10.1371/journal.pone.0023100)

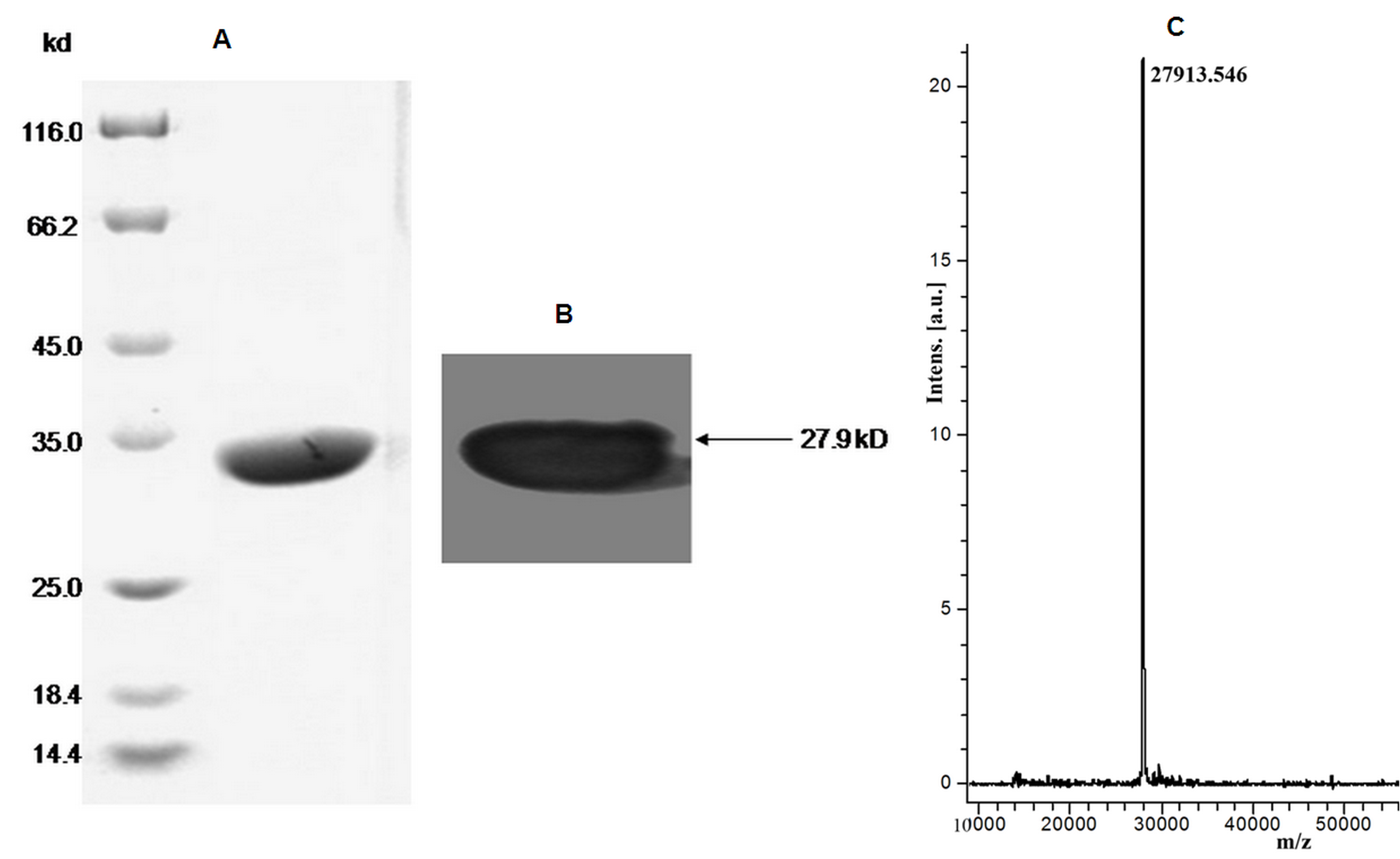

Supplement: Figure S1 — Identification of purified recombinant SF-PhoQc. (A) Purified recombinant SF-PhoQc was analyzed by SDS-PAGE. The theoretical molecular mass calculated according to the amino acid sequence of SF-PhoQc is 27.9 kDa. (B) The purified recombinant protein was confirmed by Western blot with a monoclonal antibody against the His tag. (C) The molecular mass of the protein was determined by mass spectrometry. The MS spectral data shown gives a 27.914 kDa molecular mass of the recombinant SF-PhoQc, which matches the theoretical molecular mass of 27.914 kDa calculated from the amino acid sequence. (TIF) [file pone.0023100.s001.tif]

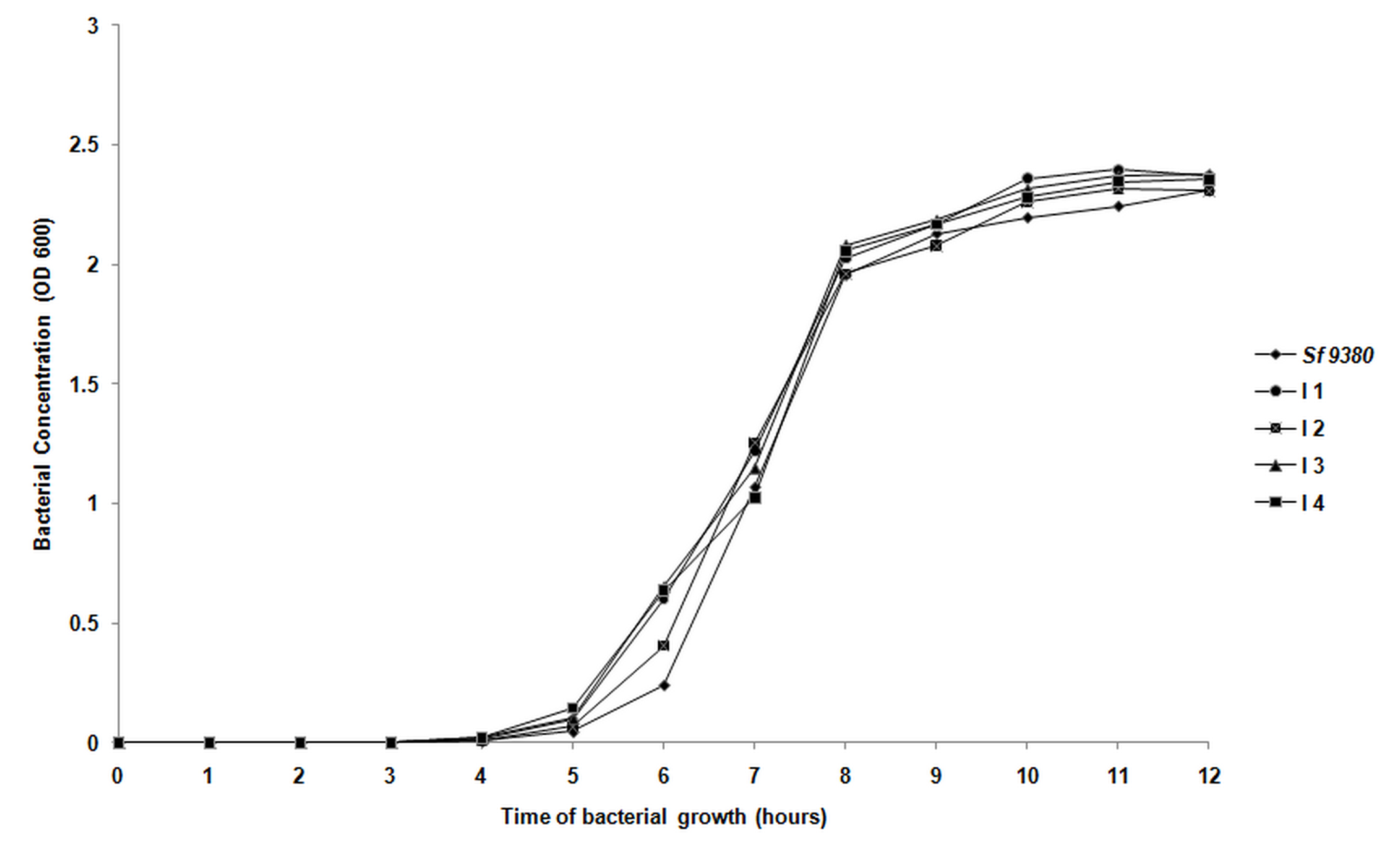

Supplement: Figure S2 — The potential PhoQ inhibitors have no effects of on Shigella growth. The growth curves of Sf9380 which treated with four potential PhoQ inhibitors were tested. Sf9380 inoculated with potential PhoQ inhibitors (final concentration 200 µmol/L), and Bacterial Concentration (OD 600) of Sf9380 in the culture were counted every one hour, for 12 hours. X-axis was the hours of bacterial growth, Y-axis was the Bacterial Concentration (OD 600). These result shows that four potential PhoQ inhibitors can not affect the growth of S. flexneri. (TIF) [file pone.0023100.s002.tif]

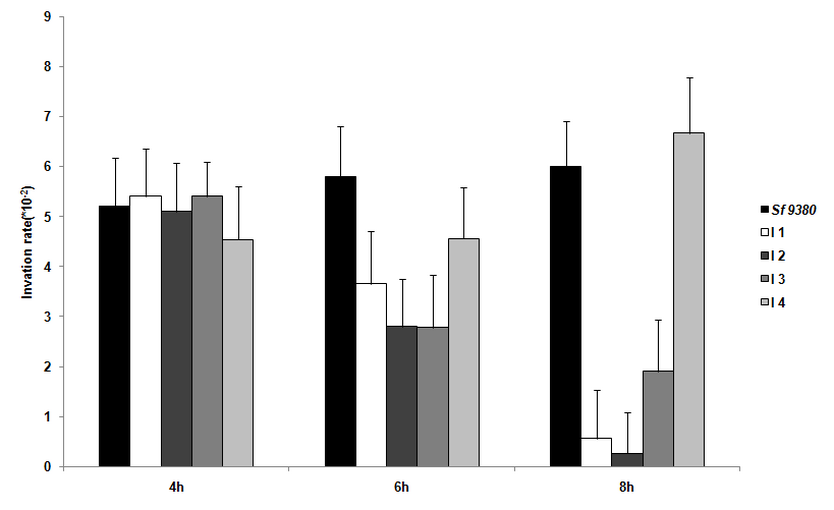

Supplement: Figure S3 — Duration of treatment influences the effects of potential PhoQ inhibitors on cell invasion of Shigella . The gentamicin protection assay shows the inhibitory effect of the four potential PhoQ inhibitors on Shigella HeLa cells invasion. The S. flexneri Sf9380 were treated with each of the four potential PhoQ inhibitors (100 µmo/L) grown for 4, 6 or 8 hours. Then the bacteria were inoculated with HeLa cells for 60 minutes prior to the addition of gentamicin to kill extracellular bacteria. After the incubation, the HeLa cells in each well were lysed in 1 ml of PBS containing 0.1% Triton X-100. The lysates were diluted and plated onto LB agar plates in triplicate. Colonies grown on LB plates were counted. The results of the assays were expressed as the number of bacteria recovered from gentamicin-treated cells divided by the number of inoculated bacteria added to the cell. Result indicated that compared with cell invasion of the positive control Sf9380 alone, the potential PhoQ inhibitors (100 µmol/L) treated for 8 hours had obvious inhibition effects on the bacteria cell invasion, while potential PhoQ inhibitors treated for 4 or 6 hours had no significant inhibition effects on Shigella cell invasion. (TIF) [file pone.0023100.s003.tif]

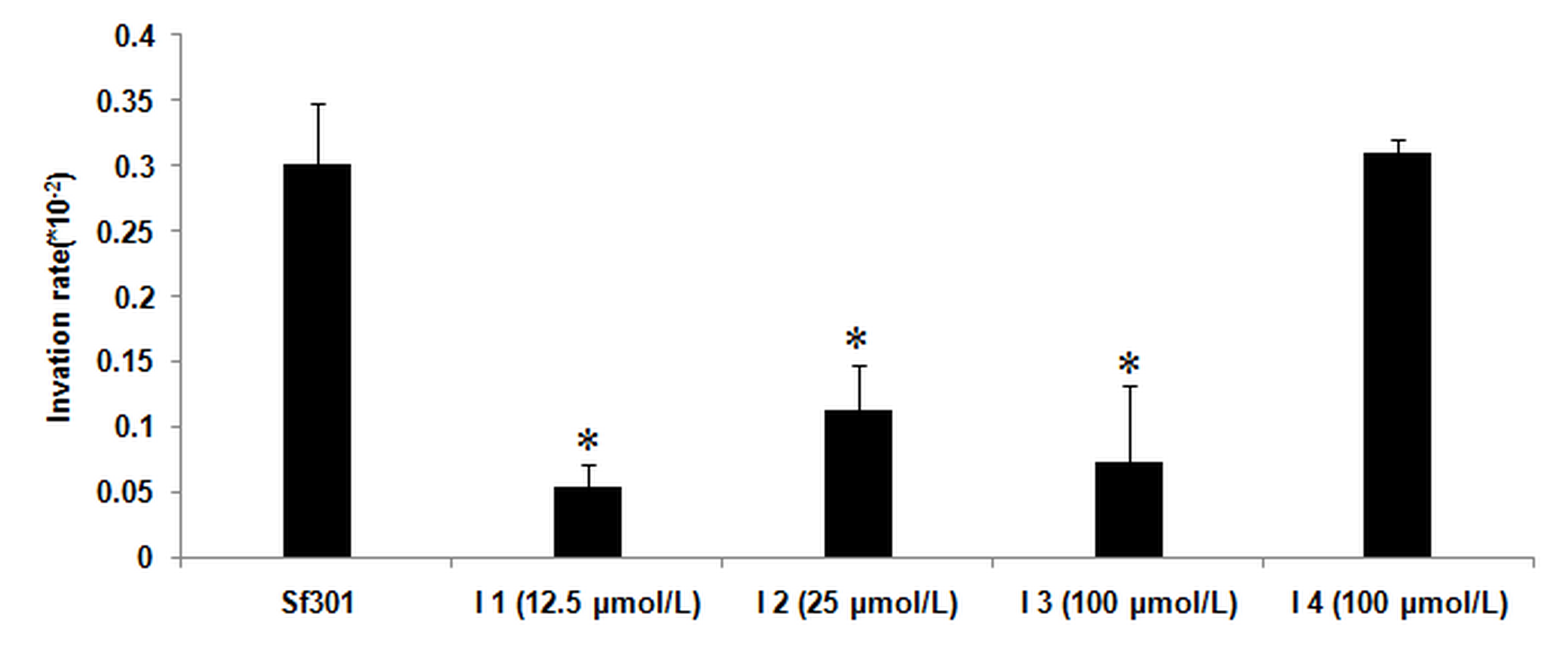

Supplement: Figure S4 — The effects of potential PhoQ inhibitors on cell invasion of Shigella Sf 301. The gentamicin protection assay shows the inhibitory effect of the four potential PhoQ inhibitors on S. flexneri 2a 301 HeLa cells invasion. The bacteria were treated with each of the four potential PhoQ inhibitors grown for 8 h. Then the bacteria were inoculated with HeLa cells for 60 minutes prior to the addition of gentamicin to kill extracellular bacteria. After the incubation, the HeLa cells in each well were lysed in 1 ml of PBS containing 0.1% Triton X-100. The lysates were diluted and plated onto LB agar plates in triplicate. Colonies grown on LB plates were counted. The results of the assays were expressed as the number of bacteria recovered from gentamicin-treated cells divided by the number of inoculated bacteria added to the cell. Values are means ± standard deviations from 6 independent wells. *p<0.01 vs. Sf301. The results indicated that potential PhoQ inhibitors 1, 2, 3 can inhibit HeLa cell invasion ability of Sf301 while potential PhoQ inhibitor 4 had no effect on Sf301 invasion of HeLa cells. (TIF) [file pone.0023100.s004.tif]
